# Internet-Based Patient Education Prior to Colonoscopy: Prospective, Observational Study of a Single Center’s Implementation, with Objective Assessment of Bowel Preparation Quality and Patient Satisfaction

**DOI:** 10.1093/jcag/gwz026

**Published:** 2019-09-04

**Authors:** Roberto Trasolini, Estello Nap-Hill, Matthew Suzuki, Cherry Galorport, Jordan Yonge, Jack Amar, Brian Bressler, Hin Hin Ko, Eric C S Lam, Alnoor Ramji, Gregory Rosenfeld, Jennifer J Telford, Scott Whittaker, Robert A Enns

**Affiliations:** Division of Gastroenterology, Department of Medicine, St. Paul’s Hospital, University of British Columbia, Vancouver, British Columbia

## Abstract

**Background:**

Nonpharmacologic factors, including patient education, affect bowel preparation for colonoscopy. Optimal cleansing increases quality and reduces repeat procedures. This study prospectively analyzes use of an individualized online patient education module in place of traditional patient education.

**Aims:**

To determine the effectiveness of online education for patients, measured by the proportion achieving sufficient bowel preparation. Secondary measures include assessment of patient satisfaction.

**Methods:**

Prospective, single-center, observational study. Adults aged 19 years and over, with an e-mail account, scheduled for nonurgent colonoscopy, with English proficiency (or someone who could translate for them) were recruited. Demographics and objective bowel preparation quality were collected. Patient satisfaction was assessed via survey to assess clarity and usefulness of the module.

**Results:**

Nine hundred consecutive patients completed the study. 84.6% of patients achieved adequate bowel preparation as measured by Boston bowel preparation score ≥ 6 and 90.1% scored adequately using Ottawa bowel preparation score ≤7. 94.2% and 92.1% of patients rated the web-education module as ‘very useful’ and ‘very clear’, respectively (≥8/10 on respective scales).

**Conclusions:**

Our analysis suggests that internet-based patient education prior to colonoscopy is a viable option and achieves adequate bowel preparation. Preparation quality is comparable to previously published trials. Included patients found the process clear and useful. Pragmatic benefits of a web-based protocol such as time and cost savings were not formally assessed but may contribute to greater satisfaction for endoscopists and patients.

## INTRODUCTION

Good bowel preparation has been associated with adenoma detection rate, detection of sessile serrated polyps and decreased presence of advanced adenomas on repeat colonoscopy (1–3). As experience with colonoscopy has grown, increasing emphasis has been placed on quality indicators such as adenoma detection rate based on associated decreases in interval cancers and cancer-related deaths (4–6). As many as 42% of adenomas and 27% of advanced adenomas may be missed with poor quality preparation (3). Poor preparation is also associated with repeat procedures, is responsible for nearly 25% of failed colonoscopies and may lead to increased complication rates (7,8).

Many trials have been performed to determine the optimal precolonoscopy diet, ideal bowel preparation compounds and administration regimens (7). Nonpharmacologic factors, however, including patient education, motivation, comorbid illness, age and cultural factors all impact quality of bowel preparation (9–15). Interventions aimed at improving patient education and motivation has shown clinically significant improvement in bowel preparation independent of regimen (16). Successful strategies have been described in the literature including brief counselling and educational pamphlets for inpatients (9,12,17), use of cartoon visual aids or online videos (10,18,19), telephone reminders (11,20) and a social media application (13). All the interventions were reasonably successful and meta-analysis shows improved rates of adequate bowel preparation from 78.4% to 88.5% for routine education compared to enhanced education methods (16).

While enhanced patient education has been shown to improve patient and endoscopist relevant outcomes, several barriers exist to uptake. Busy schedules limit the amount of time clinicians can spend counselling. Additionally, instruction varies depending on regimen, time of procedure, language and patient specific variables. An ideal educational tool should therefore be customizable, accessible to a wide range of education levels and maximize patient engagement (15). With these factors in mind our group developed an internet-based education module that is customizable for each patient with the goal of improving efficiency and efficacy of precolonoscopy education.

The module was written to be comprehensible to anyone with a sixth-grade education, incorporates education on the benefits of effective bowel preparation, cartoon visual aids and requires light user participation to maximize engagement and retention. While the current iteration was written in English, cartoon visual aids, the simple language used and the ability to send individualized links to patients would facilitate translation to other languages. Instruction in languages rarely encountered could also be feasible given the lack of a need for physical storage of educational materials. The system was tested through a pilot study of 450 patients comparing the online education module to traditional paper handouts. This initial trial showed a significant improvement in proportion of patients achieving excellent bowel preparation and a significant decrease in the number of patients with poor bowel preparation. Specifically, comparing online education to traditional paper-based education, 47% versus 37% of patients achieved a Boston Bowel Preparation Scale (BBPS) score of ≥8 (*P* = 0.036 using Fishers exact test) and 3.5% compared to 8.9% had BBPS of ≤3 (*P* = 0.019 using Fishers exact test). Patient satisfaction scores were similar with both paper and online education materials (21). Based on these data, our group transitioned to using the online module as the standard of care for patient education in our clinic and began collecting data on a larger number of patients to validate the use of the module in a pragmatic, real world setting.

## MATERIALS AND METHODS

### Study Description

This was a prospective, observational study conducted between February 2015 and September 2016. The study was institutional review board approved. No external funding was obtained. All patients who met inclusion criteria were invited consecutively from referrals for outpatient, nonurgent, screening, surveillance or diagnostic colonoscopy to the gastroenterology group at St. Paul’s Hospital, a tertiary care centre in Vancouver, Canada. Referrals included patients from the local urban area and the surrounding suburban communities and included patients from a wide range of socioeconomic, educational and ethnic backgrounds. Patients were recruited consecutively from referrals to the gastroenterology group and included colon cancer screening, iron deficiency and chronic diarrhea most commonly. Patient consent to participate and collect their data was obtained prior to enrolment. Patients were asked by a research assistant to confidentially complete satisfaction questionnaires prior to their procedures. Endoscopists were counselled on accurate scoring and asked to rate quality of preparation using both BBPS and Ottawa Bowel Preparation Scale (OBPS) on standardized forms that were completed immediately after the procedure. BBPS and OBPS scores were chosen for their validated inter-observer reliability (22). Basic patient demographics were also collected. Nine different endoscopists contributed their assessment of bowel preparation quality on 100 separate patients each. All data collected were treated similarly to routine clinical data and stored on secure, privacy legislation compliant hard drives and servers.

### Inclusion Criteria

Adults aged 19 years or greater, English proficiency or an available family member or friend willing to translate for them and a functioning e-mail account.

### Exclusion Criteria

Unwillingness to participate in the trial or previous partial colectomy. Direct to colonoscopy referrals were also excluded for logistical reasons as e-mail is often not used in this situation due to urgency or inability to obtain e-mail prior to being seen.

### Internet-Based Education Module

**Figure 1.**
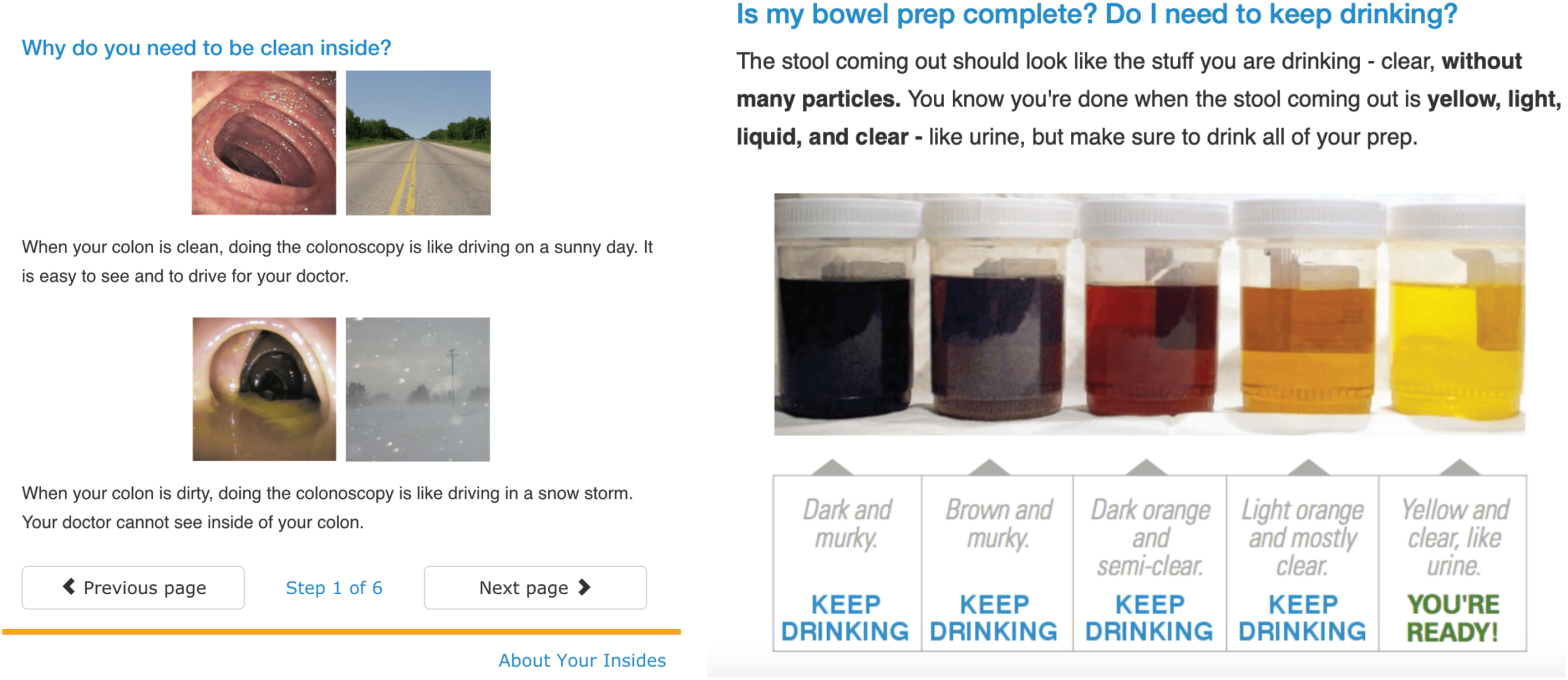
Examples of generic patient education slides.

**Figure 2.**
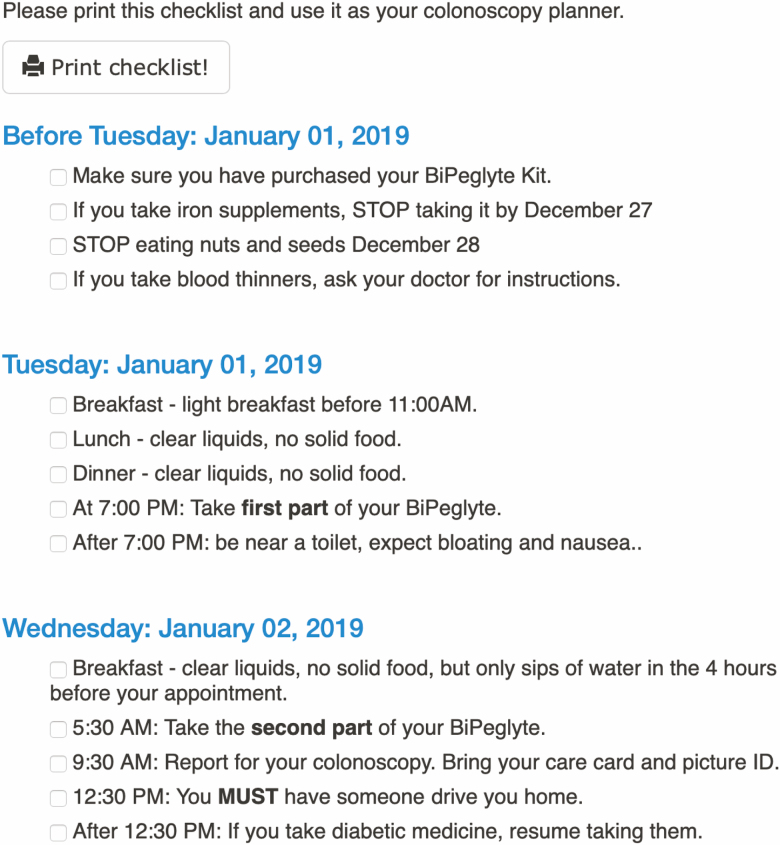
Example of individualized patient checklist from online patient education module. This slide was customized for this patient via tick-box selection and embedded in the educational module.

A customizable internet-based education module was created with drop-down menu selections made for type of preparation, split versus single dose, other prescribed medications, time and date of the procedure. To distribute the module, the endoscopist selects their preparation of choice and the planned date and time of the procedure after a preprocedure consultation. After the selections are made, a unique web link is sent to the patient’s e-mail address containing general as well as individualized instructions including specific medication and dietary recommendations displayed in a calendar format. The module was designed as a 15- to 30-minute click-through tutorial containing both images and text designed to be understandable to anyone with a sixth-grade education or higher. The module requires active participation on the part of the patient and completion, responses and time spent on the module all could be collected and viewed by the endoscopist prior to the procedure though this was not assessed as part of this study. Patient internet protocol addresses were not identifiable or stored. Modifications could be made and sent to patient in the form of a new link if needed due to schedule changes, for example. Generic (Figure 1) and customized (Figure 2) sample slides from the module can be seen in the below figures.

### Statistical Analysis

Our primary endpoint was the proportion of patients who attained an adequate preparation as measured by both BBPS and OBPS score. Adequate preparation was defined as BBPS ≥6 based on a consensus definition of adequacy from a large retrospective trial (23). OBPS score ≤7 was defined as adequate based on receiver operator curve analysis to determine the minimal cut-off at which a 5 mm polyp can be confidently diagnosed (22). Proportion of patients achieving adequate preparation as well as mean BBPS and OBPS score were calculated.

## RESULTS

Consecutive patients were invited to participate in the trial until a total of 100 patients per endoscopist agreed to participate and completed colonoscopy and data collection. Nine hundred participants in total completed satisfaction surveys and had colonoscopies with quality of bowel preparation graded. Data were not collected on patients who declined to participate or failed to complete the survey so the response rate is unfortunately not available. A post-hoc estimate by the primary research assistant based on surveying endoscopists and personal interaction with participants is that the overwhelming majority of patients had no objections to participating an estimated 50 to 75 patients refused to fill out the survey prior to colonoscopy. Typical reasons for refusal to participate included inability to speak English with no one available to translate at home and, much less commonly, lack of access to a computer or e-mail address. Typical reasons to refuse to fill out the survey prior to colonoscopy included anxiety around upcoming procedure, inability to administer survey in a timely manner prior to procedure, patient did not complete module or absence of a translator on the day of the procedure. Mean age of participants was 58 years and 469 were male (Table 1). For the primary outcome, a total of 84.6% of patients achieved an adequate preparation defined as BBPS score of ≥6. 90.1% of patients achieved an adequate preparation using OBPS score ≤7. Segment-specific scores are listed in Table 2. For the secondary outcome of patient satisfaction, 94.2% of patients scored the web-education tool as ‘very helpful’ (8 out of 10 or higher on our scale). 92.1% rated the tool as ‘very clear’ (8 out of 10 or higher on our scale).

**Table 1.** Basic demographic data of the study participants

Characteristics	
Number of patients	*N* = 900
Male Gender – *N* (percentage)	469 (52)
Age (mean)	58
Age (range)	19–82

**Table 2.** Number of patients (percentage) achieving a given Boston Bowel Preparation Score by colon segment using the web-based tutorial

Colon segment	BBPS score			
	0	1	2	3
Left	25 (3)	75 (8)	234 (26)	566 (63)
Transverse	26 (3)	72 (8)	295 (33)	507 (56)
Right	33 (4)	113 (13)	415 (46)	339 (38)

BBPS, Boston Bowel Preparation Scale.

## DISCUSSION

This prospective observational study suggests that an individualized, online, interactive bowel preparation education tool achieves high levels of adequate bowel preparation and can be a reasonable alternative compared to routine in-office education. For patients, only a brief in-office description of bowel preparation was required and an e-mailed link to the module given with no paper handouts or instructions. The module could be accessed multiple times, be re-sent if needed and paper checklists could be printed from home through the module. Despite the relatively limited in-office teaching, the patients still had high rates of quality preparation suggesting that the online method of instruction was effective and acceptable to patients. Importantly, however, results from this study are moderated by the possibility of selection bias given our lack of an empirically measured response rate.

The internet-based education module in this study used multiple enhanced education techniques, including cartoon visual aids and e-mail reminders and has the advantage of being easily adaptable to any preparation regimen by simply inputting the specifics of the regimen as an option that can subsequently be selected by the clinician or an assistant. Further customization for management of specific medications prior to endoscopy could be incorporated into the design, though the complexity of perioperative medication management would require significant customization. So far, the design has been straightforward to use and functions on android mobile devices as well as most web browsers on laptop or desktop devices. Avoiding the use of video and using only simple images was intentional for ease of use and to limit hardware and software requirements for the end-user. Both the urban location and requirement for an e-mail address in this study likely biases the included participants toward those more comfortable with computers. For groups considering an electronic patient education module, paper or in person education would likely still be occasionally necessary.

While we did not have a comparison group in this study, rates of adequate bowel preparation are grossly comparable to published trials and are compared in Table 3 to a list of trials published from 2010 to 2016 that were included in a meta-analysis on colonoscopy education techniques (16). Statistical comparison was avoided given our inability to control for bowel preparation regimen and other confounding variables. However, a prospective trial from 2015/2016 comparing this study’s online education module to traditional paper-based teaching showed a significant improvement from using the online module (21). Theoretical benefits to online education might include time savings, cost savings through reduced printing and storage, efficient use of support staff and improved consistency. Costs include those needed to develop and upkeep the software. These costs are not negligible and this type of system would probably be of most interest to groups with preference for an entirely electronic system and willing to commit to setup and maintenance costs While the current study results may not be generalizable to all populations, we hope the results of this trial provide useful ideas and useable real-world experience for groups interested in improving colonoscopy quality through patient education. Further studies with this platform are planned.

**Table 3.** Summary comparison of enhanced patient education modalities and bowel preparation quality

	Education modality	Cleanliness scores mean ± SD
Current study	Online module	OBPS 3.4, BBPS 7.0
Calderwood et al., 2011 (19)	Visual aid	BBPS 6.0 ± 0.7
Kang et al., 2016 (13)	Social Media	OBPS 3.6 ± 1.7
Lee et al., 2015 (20)	Telephone, SMS	BBPS 6.8 ± 1.3
Liu et al., 2014 (11)	Telephone	OBPS 3.0 ± 2.3
Spiegel et al., 2011 (17)	Booklet	OBPS 4.4 ±2.3
Tae et al., 2012 (10)	Cartoon	BBPS 7.4 ±1.9

BBPS, Boston Bowel Preparation Scale; OBPS, Ottawa Bowel Preparation Scale.

## Funding

No external funding was obtained for this study.

## Disclosure

R.T., E.N.-H., M.S., C.G., J.A., B.B., H.H.K., E.L., A.R., G.R., J.J.T., S.W. and R.E. have no conflicts of interest to disclose.
